# 3D Printing of Tunable Zero-Order Release Printlets

**DOI:** 10.3390/polym12081769

**Published:** 2020-08-07

**Authors:** Fabrizio Fina, Alvaro Goyanes, Martin Rowland, Simon Gaisford, Abdul W. Basit

**Affiliations:** 1 Department of Pharmaceutics, UCL School of Pharmacy, University College London, 29-39 Brunswick Square, London WC1N 1AX, UK; fabrizio.fina.14@ucl.ac.uk (F.F.); a.goyanes@ucl.ac.uk (A.G.); s.gaisford@ucl.ac.uk (S.G.); 2 Departamento de Farmacología, Farmacia y Tecnología Farmacéutica, I+D Farma (GI-1645), Facultad de Farmacia, and Health Research Institute of Santiago de Compostela (IDIS), Universidade de Santiago de Compostela, 15782 Santiago de Compostela, Spain; 3 Pfizer Ltd., Drug Product Design, Discovery Park, Ramsgate Road, Sandwich CT13 9ND, UK; martin.rowland@pfizer.com

**Keywords:** three dimensional printing, printing pharmaceuticals, personalized medicines, controlled release, 3D printed drug products, computer aided drug design and delivery, digital pharmaceutics, health and pharmaceutical sciences, gastrointestinal modified release drug delivery

## Abstract

Zero-order release formulations are designed to release a drug at a constant rate over a prolonged time, thus reducing systemic side effects and improving patience adherence to the therapy. Such formulations are traditionally complex to manufacture, requiring multiple steps. In this work, fused deposition modeling (FDM) 3D printing was explored to prepare on-demand printlets (3D printed tablets). The design includes a prolonged release core surrounded by an insoluble shell able to provide zero-order release profiles. The effect of drug loading (10, 25, and 40% *w*/*w* paracetamol) on the mechanical and physical properties of the hot melt extruded filaments and 3D printed formulations was evaluated. Two different shell 3D designs (6 mm and 8 mm diameter apertures) together with three different core infills (100, 50, and 25%) were prepared. The formulations showed a range of zero-order release profiles spanning 16 to 48 h. The work has shown that with simple formulation design modifications, it is possible to print extended release formulations with tunable, zero-order release kinetics. Moreover, by using different infill percentages, the dose contained in the printlet can be infinitely adjusted, providing an additive manufacturing route for personalizing medicines to a patient.

## 1. Introduction

For several decades, formulations that can release drugs with zero-order kinetics have been desirable for a significant number of bioactive compounds [1]. In a clinical setting, the use of repeated intravenous perfusions is a common method to maintain a constant drug concentration in the blood, especially for narrow therapeutic index drugs. However, by using this method, patient acceptability may be compromised, and the therapy can also be highly costly in the long-term [2]. Oral dosage forms are the most acceptable for patient adherence; however, in some cases immediate release tablets must be taken multiple times a day, causing plasma drug levels to fluctuate above and below the therapeutic window. To provide a constant drug plasma concentration, a formulation should be able to release the drug with zero-order kinetics. A number of approaches have been reported previously for the preparation of zero-order tablets [3,4,5,6,7,8,9,10], however these methods are complex, time-consuming, and lack flexibility (in terms of design and dose).

Three-dimensional (3D) printing is widely considered as having the potential to disrupt and transform manufacturing paradigms in the pharmaceutical sector by enabling on-demand fabrication of personalized medicines. Personalization can be in the form of dose, shape, size, color, taste, and/or release profile [11]. 3D printing could revolutionize the way oral formulations are manufactured at all stages of the drug development timeline, from preclinical studies and first-in-human (FIH) clinical trials, to on-demand production in hospital and pharmacies. The recent launch of Spritam^®^ (Aprecia Pharmaceuticals LLC) has shown that 3D printing technology can also be adapted for commercial production of oro-dispersible tablets.

In 3D printing, models are created using computer-aided design (CAD) software and the objects are fabricated by depositing materials in a layer-by-layer manner. A wide range of advanced formulations have been prepared using 3D printing ranging from oral disintegrating tablets [12], personalized oral dosage forms with innovative structures [13,14,15,16] and drug combinations [17,18,19], capsular devices [20,21,22,23], and medical devices [24,25,26,27], all of which are challenging to manufacture with traditional manufacturing methods. 3D printing has also been used in association with microfabrication to produce personalized tablets with adjustable release profiles [28,29].

3D printing of formulations with zero-order release kinetics is particularly promising. Wang et al. [1] were the first to manufacture zero-order release tablets, utilizing a powder bed inkjet 3D printing system (TheriForm process [30]). In this technology, tablets were manufactured by alternate deposition of a layer of powder and a spray of binder, repeated several times. The same technology was employed to manufacture tablets with a gradient material able to release the drug almost in a linear manner for 12–14 h [31]. In 2009, the same research group obtained an accurate zero-order release profile from donut-shaped tablets [32]. Although the results from these studies were promising, powder bed inkjet 3D printing is not a technology suited to the requirements for on-demand production of tablets in a hospital setting. In powder bed inkjet systems, the printing process and the post-treatment of the tablets (drying and excess powder removal) are time-consuming [31,32] and require staff to handle potentially toxic materials.

The manufacture of zero-order release tablets has also been investigated using semi-solid extrusion 3D printing, where a gel or paste material is extruded and solidified at room temperature [33]. In this work, the authors manufactured an insoluble membrane incorporating a pore forming excipient. Pores are formed in situ following dissolution of the pore former. The pores controlled the release of the drug (captopril) achieving a zero-order profile. A linear release profile was maintained for ~14 h, reaching 60–70% of the drug released. A different approach enclosing a conventional immediate release tablet within a 3D printed controlled-release shell was also proposed, achieving zero-order drug release profiles [34]. Semi-solid extrusion technology was recently used to conduct a clinical study in pediatric patients in a hospital setting [35]. Chewable tablets with different flavors and colors but all containing isoleucine were tailored for the treatment of a rare disease in children. Semi-solid extrusion has also been employed to print lipid-based formulations for delivering water-insoluble drugs [36], tailored levetiracetam tablets [37], and gastro-floating tablets [38].

Fused deposition modeling (FDM) is one of the most commonly used 3D printing technologies within pharmaceutical research. FDM requires filaments made of thermoplastic polymers as a feedstock material and drug can be incorporated either by impregnation [39,40,41,42,43] or hot melt extrusion (HME) [15,44,45,46,47,48,49,50]. Recently, direct powder extrusion 3D printing allowed the use of a powder blend [51,52,53] directly without extruding the filament. Previously, FDM has been investigated to manufacture zero-order release formulations. An insoluble scaffold made of acrylonitrile butadiene styrene (ABS) was printed to control the release from a conventional compressed tablet obtaining a zero-order release profile [54]. Donut-shaped tablets fabricated by FDM with a poly-vinyl-alcohol (PVA)-based core and an insoluble shell were also reported, although complete drug release took place in approximately 4 h [55]. Lately, FDM was employed to manufacture zero-order floating tablets [56,57] and a range of matrix tablets with relatively linear profiles; however, the authors did not disclose the composition of the formulations [58]. Furthermore, FDM was also investigated with different ratios of ethyl cellulose and hydroxypropyl cellulose as an easy approach to prepare zero-order release tablets for low aqueous solubility drugs [59]. However, the authors reported a drug release lower than 20% in 24 h due to the mostly insoluble tablet matrix.

The aim of the present work is to investigate FDM 3D printing to manufacture oral tablets (Printlets^TM^, 3D printed tablets) that can release tailored doses with personalized zero-order release profiles, without the need of reformulating the excipient composition. Paracetamol was selected as model drug for the present study due to its thermal stability and due to the fact that it is one of the most commonly used drugs in 3D printing studies. Establishing this technology platform will take FDM one step closer to being used to print medicines directly in a hospital or pharmacy setting. The mechanical properties of hot melt extruded filaments were evaluated to understand the effect of different drug loadings on the printability. The development of printlets with a soluble core and an insoluble release-regulating shell was investigated.

## 2. Materials and Methods

Paracetamol USP grade (Sigma-Aldrich, Gillingham, UK) was used as a model drug (BCS Class I, high solubility and high permeability, MW 151.16, solubility at 37 °C: 21.80 g/L) [60]. Hydroxypropyl cellulose (HPC, Klucel EF, MW 80 KDa, Ashland Pharmaceutics, Kidderminster, UK) was used as the thermoplastic polymer for extrusion. PEO WSR-303 NF (PEO, MW 7,000,000, Colorcon, Dartford, UK), was used as a gel matrix former. D-Mannitol (Sigma-Aldrich Co. Ltd., Gillingham, UK) was included as a plasticizer. Hydroxyethyl cellulose (HEC, Natrasol 250H, Hercules, Kidderminster UK) was included as a matrix former suspending agent. Magnesium stearate (Sigma-Aldrich Co. Ltd., Gillingham, UK) was included as a lubricant. Acrylonitrile butadiene styrene (ABS) 1.75 mm PRO PET-G white filament (RS Components, London, UK) was employed as a model material to print the shell. The salts for preparing the buffer dissolution media were purchased from VWR International Ltd., Poole, UK.

### 2.1. Preparation of Drug-Loaded Filaments by Hot Melt Extrusion (HME)

A blend of the drug and excipients (50 g) was prepared for each formulation. The components were mixed in a mortar until no visible agglomerates were observed. The composition of the formulations evaluated in this study is listed in Table 1. The theoretical drug content of the mixtures was 10, 25, or 40 % *w*/*w*. A single-screw filament extruder (Noztec Pro hot melt extruder, Noztec, Shoreham-by-Sea, UK) was used to extrude the mixture of drug and excipients in order to obtain the drug loaded filament (extrusion temperature 110–120 °C, nozzle diameter 1.75 mm, screw speed 15 rpm). The extruded filaments obtained were protected from light and stored in a vacuum desiccator until printing.

### 2.2. FDM 3D Printing

**Figure 1:**
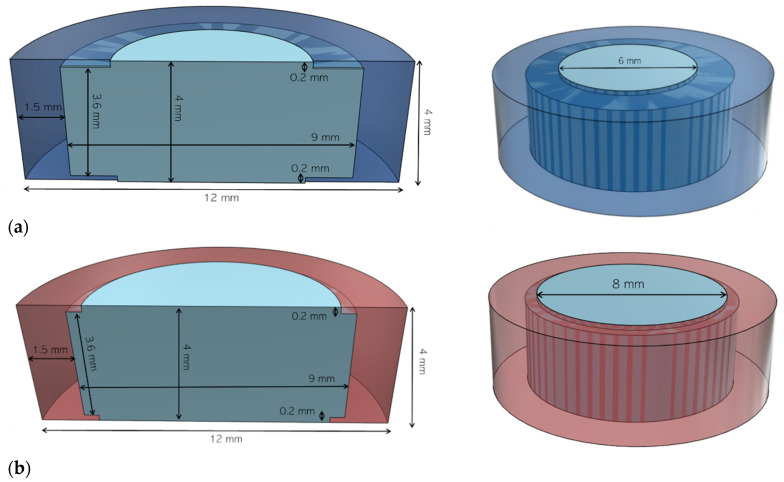
(**a**) Vertical cross section (left) and top view (right) of the 6 mm printlet 3D model; (**b**) vertical cross section (left) and top view (right) of the 8 mm printlet 3D model.

123D Design (Autodesk Inc., San Rafael, California, USA) software was used to design the templates of the printlets. Two 3D models were investigated in the present study. The first 3D model for the core (light blue) and the shell (dark blue) is illustrated in Figure 1a. Two 6 mm apertures were included, one at the top and one at the bottom of the shell. A second 3D model was designed with larger apertures (both 8 mm) to increase the dissolution rate (Figure 1b). The 3D models were then exported as a stereolithography (.stl) file into 3D printer software (MakerWare v. 3.7.0, MakerBot Inc., Brooklyn, New York, USA). The .stl format contained only the object surface data, and all the other parameters were defined from the MakerBot software in order to obtain printlets with the best resolution.

The printlets were fabricated from the extruded filaments (shell: ABS filament, core: drug-loaded filaments) using a dual-extrusion MakerBot Replicator 2X Desktop 3D Printer (MakerBot Inc., Brooklyn, New York, USA). The FDM printer settings were selected as follows; raster orientation (45°/−45° on alternate layers), standard resolution without raft and an extrusion temperature of 170 °C for the core filaments and 230 °C for the shell filament, platform temperature 80 °C, speed while extruding (90 mm/s), speed while travelling (150 mm/s), number of shells (2), and layer height (0.15 mm). Both nozzles were 0.3 mm in diameter. Infill was set at 100%, 50%, and 25% in order to produce cores with different densities. The hexagonal infill printing path was selected. Purging walls option was activated (recommended for dual extrusion, more material was required but provided a better quality).

### 2.3. Mechanical Characterization of Filaments

#### 2.3.1. Tensile Test

A mechanical tester, 5567 (Instron, Buckinghamshire, High Wycombe, UK), was used to measure the pulling force required to break the core filaments. Filaments with an average diameter approximately 1.75 mm and 40 mm gauge length were selected. The diameter of the samples (measured using a digital calliper) for various sections and the average (~1.75 mm) was programmed into the software (Bluehill 2, Version 2.35, High Wycombe, UK). The tensile extension was set to 5 mm/min and the data were collected every 50 ms. Sandpaper was used abrade the ends of each sample to prevent slippage from the clamps. All formulations were measured in triplicate. The maximum load at the breaking point and the Young’s modulus were measured. Data were statistically analyzed by performing one-way ANOVA with post hoc Tukey’s test using Origin Pro 2018 software (version b9.5.0.193, Stoke Mandeville, UK). *p*-values <0.05 were considered statistically significant.

#### 2.3.2. 3-Point Bending Test

A CT-5 tester (iHolland limited, Nottingham, UK) was employed to perform a 3-point bending test to evaluate the fracture toughness of the filaments. The CT-5 instrument measured the breaking force (F) of the filament. The fracture tensile strengths were calculated using Equation (1):

(1) $$\sigma_{T}=\frac{3}{2}\frac{F\times S}{W\times t^{2}}$$

where $\sigma_{T}$ is the fracture tensile strength (MPa), *F* is the load applied at fracture (*n*), *W* is the mean width of the sample (mm), *S* is the distance between the lower supports (mm), and *t* is the mean thickness of the sample (mm).

As the samples were cylindrical filaments, both *W* and *t* represented the diameter of the filament. The CT-5 test speed was set at 0.42 mm/s. The distance between the lower supports was selected as 15 mm. Three replicates for each sample were tested. Data were statistically analyzed as described in Section 2.3.1.

#### 2.3.3. Nanoindentation

Nanoindentation was employed to measure the hardness (resistance to penetration) of the filaments. Filament strands (~1 cm) were transferred to a glass slide and held in place with a nonviscous adhesive (Permabond). The glass slide was then attached to a metallic sample holder using a small amount of the same adhesive. The test was performed using a nanotester 600 (Micro Materials Ltd., Wrexham, UK). The nanotester continuously recorded the penetration depth of the sharp indenter as a function of the applied force throughout a loading–unloading experiment. The indenter was a diamond three-sided pyramid (Berkovich) able to move freely around an essentially frictionless pivot. Ten indentations were performed on the surface of the filament. A minimum distance of 50 µm was set between each indent. The indenter load ranged between 0.05 and 500 mN and the depth between 20 and 5000 nm, while typical resolution for the instrument was 100 nN and 0.1 nm for load and depth, respectively. After contact between the indenter tip and the filament surface, indentation was performed until a 15,000 nm depth was reached. The peak load was then held for 10 s and then the indenter was unloaded down to zero. These provided a record of the load and displacement throughout the test and the plots were analyzed to calculate the required mechanical properties. Hardness values were determined from the software.

### 2.4. Mechanical Characterization of the Printlets

#### 2.4.1. Determination of Printlet Strength

The breaking force of printlets of each type was measured as in a previous study [61]. Samples were tested in triplicate using a traditional tablet hardness tester TBH 200 (Erweka GmbH, Heusenstamm, Germany), whereby an increasing force was applied perpendicular to the printlet axis until the printlet fractured.

#### 2.4.2. Determination of Printlet Friability

The friability of printlets was measured as in a previous study [61]. Samples of printlets (approximately 6.5 g) were weighed and placed into the drum of a Friability Tester Erweka type TAR 10 (Erweka GmbH, Heusenstamm, Germany). The speed of the drum was set at 25 rpm for 4 min and the sample was reweighed after the rotation. The friability of the printlets was calculated in terms of weight loss, expressed as a percentage of the initial sample weight.

### 2.5. Thermal Analysis

Differential scanning calorimetry (DSC) measurements were performed with a Q2000 DSC (TA instruments, Waters, LLC, New Castle, Delaware, USA) at a heating rate of 10 °C/min to characterize the powder blends, filaments, and cores. A preheating cycle was used (0 °C–110 °C) to remove water of the samples. Calibration for cell constant and enthalpy was performed as in a previous study [62]. TA aluminium pans and pin-holed hermetic lids (T_zero_) were used with an average sample mass of 8–10 mg. A nitrogen purge was used with a flow rate of 50 mL/min for all the experiments. Data were collected with TA Advantage software for Q series (version 2.8.394) and analyzed using TA Instruments Universal Analysis 2000. All melting temperatures are reported as extrapolated onset unless otherwise stated. Thermogravimetric analysis (TGA) analysis was conducted on a Discovery TGA (TA instruments, Waters, LLC, New Castle, Delaware, USA). Each sample of excipients and filaments with an average weight of 4–7 mg was heated at 10 °C/min in open aluminium pans using nitrogen as a purge gas of 25 mL/min. Data collection and analysis were performed using TA Instruments Trios software (New Castle, Delaware, USA) and % mass loss and/or onset temperature were calculated.

### 2.6. X-ray Powder Diffraction (XRPD)

Discs (23 mm diameter × 1 mm height, 100% infill) made from drug-loaded polymer filaments were 3D printed and analyzed together with samples of pure paracetamol; pure polymers (HPC and HEC); and filaments F10, F25, and F40. The XRPD patterns were obtained as in a previous study [62] in a Rigaku MiniFlex 600 (Rigaku, The Woodlands, Texas, USA) with a Cu Kα X-ray source (λ = 1.5418 Å). The intensity and voltage applied were 15 mA and 40 kV, respectively. The measurements were collected over a range of 2θ = 3–60° with a stepwise size of 0.02° at a speed of 5°/min.

### 2.7. Morphology Characterization

The physical dimensions of the printlets were measured using a digital caliper. Pictures of the filaments and printlets were taken with a Sony α6300 camera (London, UK).

### 2.8. Scanning Electron Microscopy (SEM)

Surface and cross sections of the extruded filaments were imaged by scanning electron microscopy (SEM). All samples were placed on double-sided carbon tape, mounted on stubs, and sputter coated using a Polaron E5000 with Au/Pd for 1 min prior to imaging. The stub was then placed into a Philips XL30 FEG SEM operating at 20 kV to obtain the images.

### 2.9. Determination of Drug Loading

A core (~300 mg) was placed in a 200 mL volumetric flask of deionized water, followed by magnetic stirring until complete dissolution (*n* = 2). Samples of solutions were then filtered through 0.45 µm filters (Millipore Ltd., Dublin, Ireland) and the concentration of drug determined with high-performance liquid chromatography (HPLC) using a method described in a previous study [62].

### 2.10. Dissolution Studies

The in vitro dissolution tests were performed using a USP-II apparatus (Model PTWS, Pharmatest, Hainburg, Germany). In each assay, the printlets were placed at the bottom of the vessel in 0.1 M HCl (pH 1.2, 750 mL) for 2 h under constant paddle stirring (50 rpm) at 37 °C. The printlets were then transferred to a phosphate buffer (pH 7.4, 900 mL). During the dissolution test, samples of solution were automatically removed and filtered through 10 μm filters and the drug concentration was determined using an in-line UV spectrophotometer (Cecil 2020, Cecil Instruments Ltd., Cambridge, UK) operated at the wavelength of maximum absorbance of the drug in 0.1 N HCl (247 nm). Data were processed using Icalis software (Icalis Data Systems Ltd., Berkshire, UK) and reported throughout as mean ± standard deviation. Tests were conducted in triplicate under sink conditions.

The drug release profile kinetics were analyzed by fitting the experimental data to four mathematical models including zero-order (Equation (2)), first-order (Equation (3)), Higuchi (Equation (4)), and Korsmeyer–Peppas (Equation (5)) using Excel software (Microsoft Office 365, London, UK) [63,64,65]. The correlation coefficient (R^2^) was determined to evaluate which model is the most appropriate for the drug release.

(2) $$lnQ=k_{0}t$$

(3) $$lnR=k_{1}t$$

(4) $$Q=k_{H}\sqrt{t}$$

(5) $$logQ=logk_{p}+nlogt$$

where *Q* is the percentage of cumulative drug released over time *t*; *R* is the percentage of cumulative drug remaining; *n* is the exponent of release; and *k_0_*, *k_1_*, *k_H_*, and *k_p_* are the Higuchi rate constants of zero-order, first-order, Higuchi, and Korsmeyer–Peppas models, respectively.

## 3. Results

**Figure 2:**
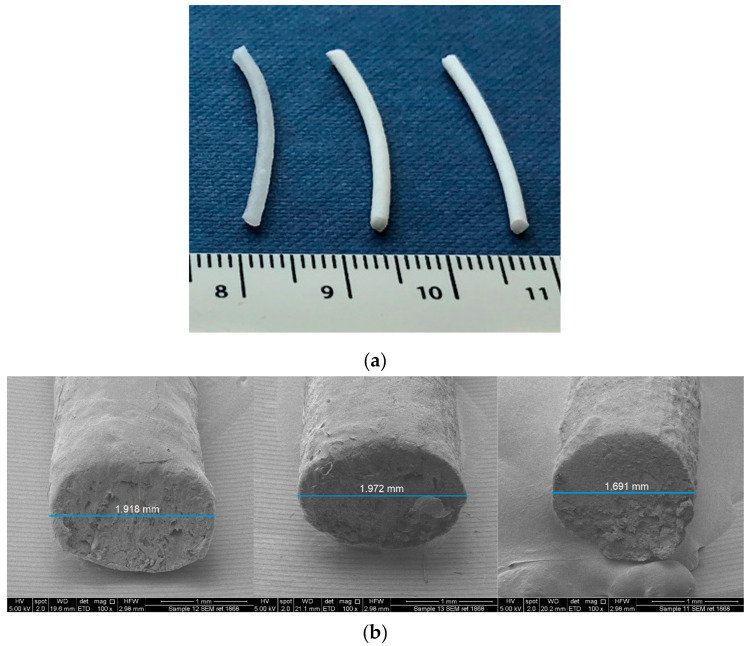
(**a**) Picture and (**b**) SEM images of core filaments with different drug loadings. From left to right: F10, F25, and F40.

Three paracetamol-loaded filaments (10, 25, 40% *w*/*w*) were successfully prepared using hot melt extrusion (Table 1; Figure 2). Three percent *w*/*w* magnesium stearate was included as a lubricant in the formulations to facilitate the extrusion process. The filament composition included hydroxypropyl cellulose (HPC) as a main thermoplastic polymer. Klucel EF was employed in the present study to provide a longer sustained release of the drug [66]. Polyethylene oxide 7,000,000 Da (PEO) was selected for this study to prolong the release and to generate a swellable hydrogel where the drug can be suspended. Hydroxy ethylcellulose (HEC) 250H was included at 5% *w*/*w* as a suspending agent. Mannitol, being a highly water-soluble sugar, was included as a pore former and plasticizer [67]. Figure 2 shows the extruded filaments and the corresponding SEM images with their measurements. F10 and F25 diameters were higher compared to F40 because the filaments with lower drug loadings were more prone to deform after cutting for SEM analysis. Only F40 retained its shape after cutting resulting in a circular cross section profile. As the drug loading increased, the filaments exhibited a proportionally smoother surface finish and a more pronounced white color.

### 3.1. Mechanical Characterization of the Filaments

#### 3.1.1. Pulling Tensile Strength

An investigation into the mechanical properties of the filaments was conducted to provide understanding on the potential printability of the filaments. Pulling tensile strength (tensile test) measurements were performed on the core filaments (Table 2). The two ends of the filaments were stretched apart until the breaking point. The maximum load at break was then recorded (Table 2). A proportionally increased maximum load was observed with the increasing drug content (F10 < F25 < F40). All the data showed a *p*-value lower than 0.05, indicating that the data were statistically significant (overall ANOVA). Comparison between groups (Tukey’s test) showed the tensile strengths of F10-F40 and F25-F40 were significantly different. ABS was also evaluated in terms of pulling tensile strength. However, the ABS samples were impossible to break, and slipped out of the instrument’s clamps during testing and so no values could be obtained.

Materials with a high Young’s modulus are stiff and exhibit minimal mechanical deformation under elastic loads (e.g., diamond). On the other hand, materials with a low Young’s modulus are flexible and change their shape considerably (e.g., rubbers). Interestingly, by increasing the amount of drug (F10, F25, and F40), the Young’s modulus notably increased (Table 2) and reached almost 150 MPa for F40. As for tensile strength, the Young’s modulus values were significantly different between F10-F40 and F25-F40, but not were not significantly different between F10 and F25. The drug acts as plasticizer at lower concentrations (10–25% *w*/*w*); however, at 40% *w*/*w*, the solubilizing effect of the polymer is saturated and most of the drug remains suspended in crystalline form increasing the stiffness of the filament.

#### 3.1.2. Flexural Tensile Strength

A 3-point bending test was employed to measure the flexural tensile strength of the filaments [48,68]. Brittleness measurements were performed for the shell filament (ABS) and the drug-loaded filaments (Table 2).

ABS is provided as a commercial filament with ideal mechanical properties, and exhibited a high tensile strength of 49.1 MPa, with samples being difficult to fracture. On the other hand, drug-loaded filaments showed a much lower tensile strength that increased slightly with the higher drug content. F10 had the lowest value (4.4 MPa), followed by F25 (4.6 MPa) and F40 (5.8 MPa). The tensile strengths were significantly different between F10-F40 and F25-F40, but not were not significantly different between F10 and F25. Although the values were similar, filament F40 exhibited the best qualities for printing. These data confirmed that higher fracture tensile strength values were likely to increase the printability of the filament.

#### 3.1.3. Indentation Hardness

**Figure 3:**
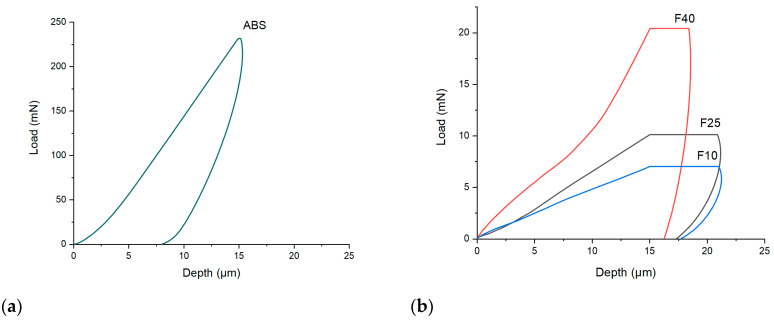
(**a**) Nanoindentation analysis of the ABS filament; (**b**) nanoindentation analysis of the core filaments with different drug loading: F10 (10% *w*/*w* paracetamol), F25 (25% *w*/*w* paracetamol), and F40 (40% *w*/*w* paracetamol).

Filaments were characterized in terms of hardness using a nanoindenter. Hardness values can help to predict the printability of the filaments together with the other mechanical measurements [69]. Results for the ABS filament (Figure 3a) showed that ~250 mN was necessary to indent the surface of a filament down to 15 µm in depth. Once 15 µm was reached, the load was held constant for 30 s to evaluate any further penetration. Almost no further penetration was recorded, indicating that the filament was very tough. 

Load values were then measured for the filaments F10, F25, and F40 (Figure 3b). With the increasing drug loading, increased load was required to achieve a 15 µm penetration of the indenter. Interestingly, no clear difference could be observed between the filaments F10 and F25, with 7 and 10 mN, respectively, while the filament with 40% *w*/*w* drug content (F40) required over 20 mN for the sample to be penetrated. Additionally, during the 30 s hold, the indenter further penetrated F40 for only ~3.5 µm (the horizontal line of F40), compared with ~6.0 µm obtained from F25 and F10. These results confirmed the higher hardness of F40 compared to F10 and F25. From the load values, the hardness of the filaments was therefore calculated from the software (Table 2). The hardness value confirmed as F40 with 40% *w*/*w* paracetamol was mechanically different from F10 and F25 that reported very similar properties.

### 3.2. Physical Characterization

**Figure 4:**
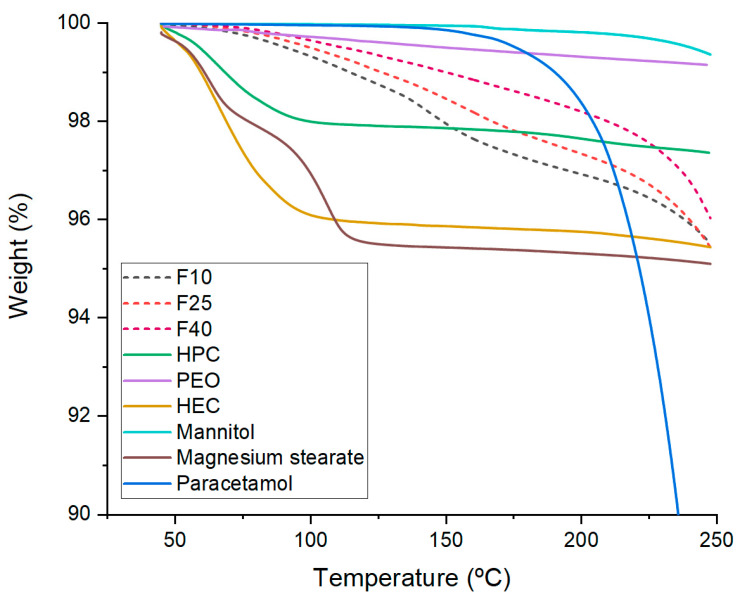
TGA of the filaments, drug, and individual excipients included in the compositions.

TGA was performed for the filaments with different drug loadings (F10, F25, and F40) to assure their stability at printing temperature (Figure 4). Core filaments (F10, F25, and F40) also showed over 95% of the total weight at printing temperature (170 °C), indicating the suitability of these compositions for FDM 3D printing.

F10, F25, and F40 were then evaluated in terms of printability. Cylindrical cores (9 mm diameter × 4 mm height) were printed with all three filaments (cores named C10, C25, and C40, respectively). Although all filaments were printable, the best printing results were obtained with F40. To achieve an acceptable printability, first the filaments need to show good feedability to allow the smooth and continuous access of the filaments to the heated section of the printing head [70]. In the FDM printer, a pinch roller system (2 rollers) is used to maintain the filament in tension. Below the rollers the filament is in compression and it is pushed inside a constricted opening of the heated printing head [71]. Filaments loaded with 10 and 25% paracetamol were quite ductile and underwent occasional squeezing between the rollers during the printing process, causing interruption or inappropriate feeding. Ductile filaments are also likely to undergo buckling before entering the printing head due to excess compression leading to printing failure. On the other hand, F40 did not present these drawbacks, showing a smooth and continuous printing process. Although possessing the same dimensions, cores printed with F40 were heavier (270 ± 5.2 mg) compared with F25 (258 ± 8.4 mg) and F10 (250 ± 15.2 mg). The lower weight indicated that even in cases where the printing process reached completion, the inappropriate feeding rate because of the properties of the filaments led to the presence of unwanted gaps inside. Additionally, the increasing standard deviation showed that the printing process with F10 and F25 was more variable compared with F40. These observations correlate with the mechanical characterizations of the filaments. As the drug loading of the filament increased, the hardness and the tensile strength (pulling and flexural) of the filaments increased, resulting in a better performance of the filament during the printing process (better grip of the filament between the pushing wheels), and consequently a higher printing quality.

**Figure 5:**
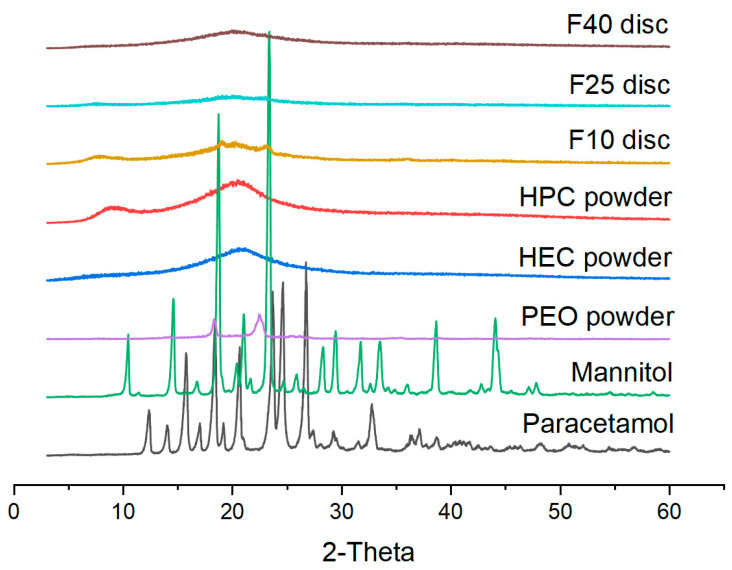
XRPD diffraction pattern of the printed formulations discs, the powdered polymers, and the drug.

**Figure 6:**
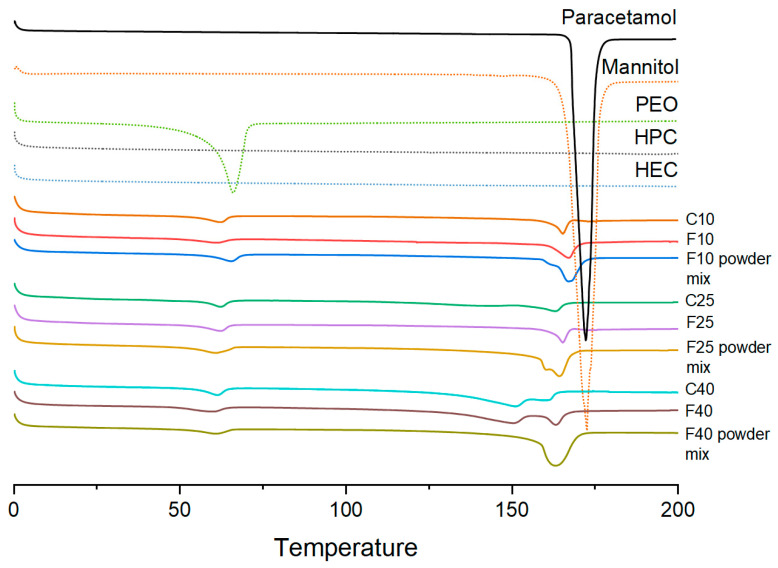
DSC analysis of individual polymers, the powder mixtures, extruded filaments (F10, F25, F40), and printed cores (endothermic events plotted downwards).

XRPD and DSC analyses were performed to evaluate in which form the drug was incorporated in the filaments and cores (Figure 5 and Figure 6). XRPD data showed broad halos for F40 and F25 discs indicating that the drug was incorporated in an amorphous form. F10, containing a higher amount of HPC (62% *w*/*w*), showed a diffraction pattern similar to HPC powder, with some small peaks at 18 and 23°, indicating the presence of either mannitol and/or PEO in a crystalline form. The presence of crystalline material only in the F10 disc can be explained with the lower extrusion temperature (110 °C compared with 120 °C for the other two filaments) or by the plasticizing effect of the increasing drug loading that might have contributed to better solubilization of mannitol and/or PEO in F25 and F40. DSC analysis (Figure 6) showed small endothermic peaks at 70 °C for all the printed formulations, indicating that part of PEO was still in a crystalline form after extrusion and printing. In all the powder mixtures, a broader peak (composed of two peaks) was present at 160–165 °C, indicating that both paracetamol and mannitol were crystalline. However, only a small endothermic peak was present at 170 °C in all the filaments indicating that the drug became amorphous after extrusion. Only in F40 was a second endotherm still present (shifted at 150 °C), indicating that a small amount of the drug was still in crystalline form but could not be detected by XRPD (F40 disc showed a complete amorphous diffraction pattern, Figure 5).

### 3.3. FDM 3D Printing and In Vitro Dissolution Tests

**Figure 7:**
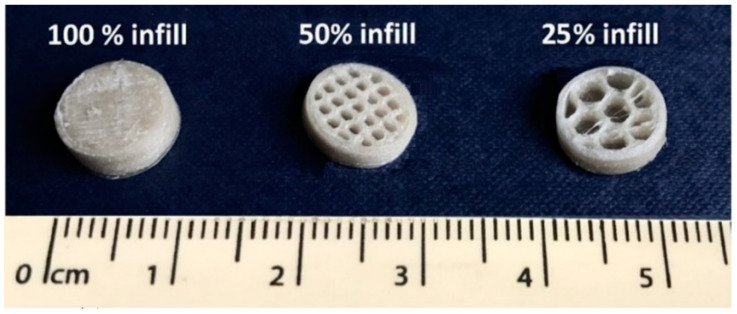
Horizontal cross sections of C40 with three infill percentages (100, 50, and 25% *v*/*v*).

The filament with the highest drug loading (F40) exhibited the most optimal mechanical properties and was therefore selected for the manufacture of printlets. Three infill percentages (100%, 50%, and 25%) were selected to explore the feasibility of controlling the release profiles. Cores were successfully printed at each respective infill (Figure 7) with a consistent diameter of 9.0 ± 0.1 mm. HPLC analysis of the drug loading of the cores showed the absence of drug degradation with an assay value of 39.4 ± 0.4%. This was in alignment with a previous study where paracetamol was proved to be stable at similar operational temperatures for FDM printing [72].

To achieve zero-order release, an insoluble shell was 3D printed with ABS onto the core to provide a rate-limiting barrier. ABS filament was selected for its high toughness and water resistance that provide a shell able to withstand long dissolution tests without deformation [73]. A similar 3D model was successfully able to release drug linearly using inkjet 3D printing [32]. Two shell models (Figure 1) were designed with 6 mm and 8 mm size apertures, respectively (Table 3). The printlets (P) were identified with the first number indicating the aperture diameter and the second number indicating the percentage of infill.

**Figure 8:**
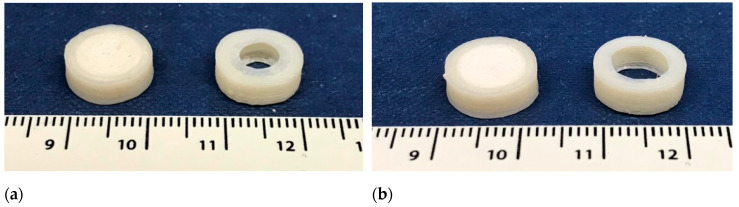
(**a**) P6-100 before (left) and after (right) the dissolution test; (**b**) P8-100 before (left) and after (right) the dissolution test. Ruler in cm.

All printlets were successfully printed using dual-extrusion FDM (one filament for the core and one for the shell). To improve the quality of the printlets, purging walls were used to avoid interaction between the two molten materials during the printing process [74]. Each printlet was fabricated in 9–12 min depending on the infill percentage, the higher the infill, the slower the process. The printlets that were prepared were physically robust and maintained the required geometry (Figure 8) with a diameter very close to the 3D model designed (Table 3). Mechanical characterization of the printlets showed ideal properties. The breaking force for the printlets was not recorded during testing, indicating that the real value was higher than the maximum limit of the instrument (485 N). Friability testing showed no physical attrition after the test. These results corroborate with previous works where FDM tablets showed a robust nature [75].

**Figure 9:**
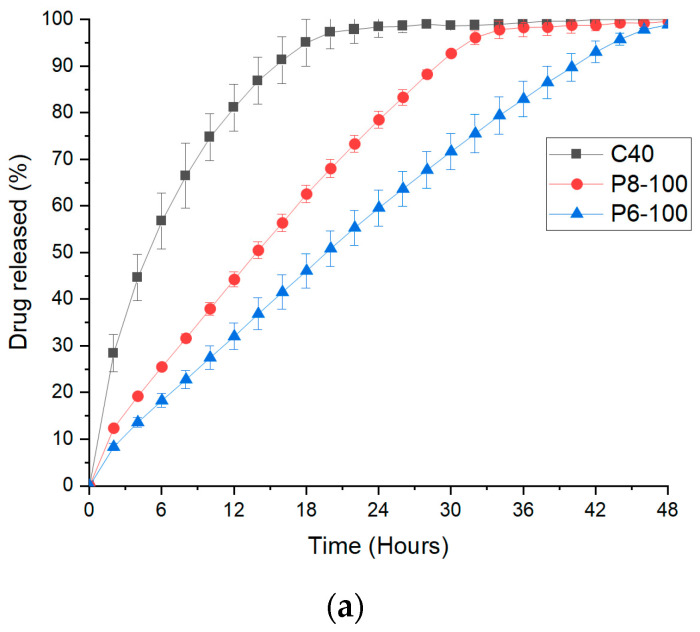

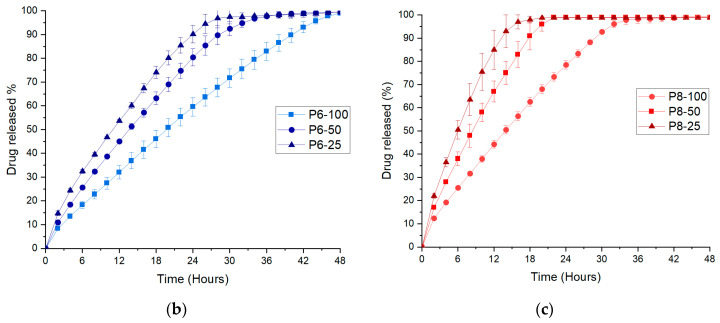
(**a**) Dissolution profiles of the core only (C40), compared with P6-100 and P8-100; (**b**) dissolution profiles of P6-100, P6-50, and P6-25; (**c**) dissolution profiles of P8-100, P8-50, and P8-25.

In vitro dissolution tests for P6-100 exhibited 100% release within 48 h, while P8-100, due to the larger aperture diameter (8 mm), achieved complete drug release in 32 h (Figure 9a). For comparison, the dissolution of only the core (C40) was evaluated and showed a first-order release profile reaching 90% drug release in ~16 h. The prolonged release of the cores was primarily due to the inclusion of PEO. PEO is a fast hydrating polymer, generating hydrogels able to control the drug release [76]. In particular, high molecular weight PEO swells to a greater extent and tends to form (when hydrated) stronger gels less liable to erode [77]. When the dissolution medium encounters the core, it will dissolve both sugar (mannitol) and water-soluble polymer (HPC), leaving empty spaces inside. Subsequently, the presence of empty spaces allows PEO to swell and create a viscous environment where the drug can be suspended and slowly diffuse out. To prevent drug sedimentation, the authors of [9] investigated a number of polymers in terms of their suspending properties. Results suggested that Hydroxy Ethylcellulose (HEC, Natrosol 250H) between 3 to 6% *w*/*w* was an optimal suspending agent. Therefore, HEC was included at 5% *w*/*w* in the core composition in the present work.

To evaluate the drug release mechanism of the printlets, four different kinetic models were employed including zero-order, first-order, Higuchi, and Korsmeyer–Peppas (Table 4).

The core only (C40) showed good correlation with a first-order release profile (0.9827), while the zero-order correlation coefficient was rather low (0.8541). When the insoluble shell was added to the core, a zero-order release kinetic was achieved for both P6-100 and P8-100 with R^2^ of 0.9945 and 0.9918, respectively.

To widen the release profiles of the printlets, the cores were printed with two other infill percentages (50 and 25%) (Figure 9b,c). Visually, printlets with different infill percentages exhibited a similar visual appearance; however, dissolution profiles for P6-50 and P6-25 (Figure 9b) showed a reduction in the dissolution time proportionally with the reduced infill percentage (lowering the core infill percentage produced cores with larger void spaces). P6-50 and P6-25 exhibited complete drug release in 34 and 28 h, respectively. Changing the infill has been adopted in several studies [44,78] to modulate the drug release. The inclusion of empty spaces inside allowed the medium to access a higher surface area of the core within the same time reducing the dissolution time. Therefore, different infills can be used to personalize the release time. Additionally, increasing the infill produced cores proportionally larger in mass (Table 3), indicating that the infill represented another valid strategy to personalize the dosage form. As expected, printlets with larger apertures in the shell (8 mm) showed reduced dissolution times compared with 6 mm printlets (Figure 9c). Dissolution tests for P8-50 and P8-25 showed drug release in 20 and 16 h, respectively. A similar finding was obtained by the authors of [54], who showed how the aperture size in an FDM-printed shell can modulate the release of the drug of a conventional (crushed) tablet.

Interestingly, the change in infill percentage did not change the zero-order nature of the printlets. The R^2^ values for the zero-order release kinetic were only moderately lower compared to the 100% infill printlets (Table 4), indicating that the increasing presence of empty spaces inside the printlet provided a slightly less constant release of the drug. Most of the printlets were also well described by the Korsmeyer–Peppas model (Table 4). Their *n* (diffusion exponent) values ranged from 0.612 to 0.674. The value of *n* is an indicator for the drug release mechanisms. Accordingly to previous studies, *n* in the range of 0.425 to 0.500 indicates that drug diffusion is the dominant release mechanism, while values above 1.0 indicate that the main mechanism is either polymer relaxation, tablet erosion, or polymer dissolution [63]. In the present work, the intermediate *n* values suggested a combination of both mechanisms. Specifically, the inclusion of high molecular weight PEO (7,000,000 Da) created a swellable hydrogel that dissolved slowly allowing the drug to be release by both diffusion and polymer dissolution. Similar *n* values have been reported in previous studies for 3D printed tablets with zero-order release profiles [34,59].

The present work demonstrated the potential of FDM 3D printing to manufacture zero-order release formulations. As the infill percentage can be precisely controlled from 100 to 0% and the size of the aperture can also be varied, it would be possible to prepare numerous dosage forms with a personalized release time using this method.

## 4. Conclusions

FDM dual-extrusion 3D printing allowed the manufacture of printlets with zero-order release profiles. Core filaments including HPC (extended release polymer) and PEO (expandable hydrogel) were successfully extruded with three drug loadings (10, 25, and 40% *w*/*w* paracetamol). Mechanical characterization of the filaments allowed the relationship between drug loading and physical robustness to be determined, which in turn provided a predictive measure of printability. Filaments with a drug loading of 40% *w*/*w* were selected to print controlled release cores. During in vitro dissolution assessment, the core composition formed a hydrogel that allowed the drug to follow a multi-diffusional pathway, resulting in a constant release rate of the drug from the printlets. An insoluble shell with apertures was added to the cores to achieve zero-order release profiles. The combination of different shell aperture diameters (6 or 8 mm) together with different core infills (100, 50, and 25%) produced a range of zero-order release profiles (spanning from 16 to 48 h). By incorporating different infill percentages and adjusting the dose in tandem, a total degree of personalization for the patient may be realized.

## Figures and Tables

**Table 1:** Compositions (% *w*/*w*) of the hot melt extruded filaments.

Filaments	HPC	HEC	PEO	Mannitol	Magnesium Stearate	Paracetamol	Extrusion Temperature (°C)
F10	62	5	10	10	3	10	110
F25	47	5	10	10	3	25	120
F40	32	5	10	10	3	40	120

**Table 2:** Properties of the filaments.

Filament	Maximum Load at Break (*n*) **	Young Modulus (MPa) **	Fracture Tensile Strength (MPa) **	Hardness (MPa)
F10	10.7 ± 0.2	72.5 ± 14.0	4.4 ± 0.1	0.87 ± 0.07
F25	11.55 ± 0.1	82.4 ± 9.9	4.6 ± 0.3	1.20 ± 0.09
F40	14.4 ± 1.1	145.3 ± 11.9	5.8 ± 0.2	4.52 ± 0.12
ABS	*	*	49.1 ± 1.2	56.23 ± 4.6

* not recorded ** *p*-value < 0.05.

**Table 3:** Characteristics of the printlets.

Printlets	Aperture Diameter (mm)	Infill (%)	Weight (mg ± SD)	Diameter (mm ± SD)
P6-100	6	100	473 ± 4.3	12.03 ± 0.04
P6-50	6	50	393 ± 7.8	12.06 ± 0.07
P6-25	6	25	333 ± 9.5	12.06 ± 0.15
P8-100	8	100	475 ± 4.6	12.04 ± 0.14
P8-50	8	50	395 ± 8.1	12.09 ± 0.12
P8-25	8	25	335 ± 8.7	12.07 ± 0.12

**Table 4:** Statistical parameters of the printlets drug release data after fitting them with different kinetic models.

Printlet	Zero-Order R^2^	First-Order R^2^	Higuchi R^2^	Korsmeyer–Peppas	
				R^2^	*n*
**Core only (C40)**	0.8541	0.9827	0.9806	0.9903	0.641
**P6-100**	0.9945	0.9054	0.9626	0.9661	0.612
**P6-50**	0.9882	0.9346	0.9664	0.9821	0.623
**P6-25**	0.9812	0.9150	0.9758	0.9948	0.626
**P8-100**	0.9918	0.9003	0.9649	0.9862	0.617
**P8-50**	0.9869	0.9063	0.9664	0.9953	0.647
**P8-25**	0.9691	0.9327	0.9755	0.9987	0.674
